# Comparison of Marginal Adaptation in Lithium Disilicate Crowns Using Three Fabrication Techniques: An In Vitro Study

**DOI:** 10.7759/cureus.101228

**Published:** 2026-01-10

**Authors:** Asmita Sodhi, Pradeep Bansal

**Affiliations:** 1 Department of Prosthodontics, Sodhi Dental Clinic and Implant Centre, Faridkot, IND; 2 Department of Prosthodontics, Dasmesh Institute of Research and Dental Sciences, Faridkot, IND

**Keywords:** adaptation, ceramic, computer-aided design, marginal gap, press

## Abstract

Introduction

The durability of all-ceramic crowns in clinical settings is fundamentally influenced by the precision of their margins, as significant marginal discrepancies can lead to the dissolution of cement, microleakage, the development of secondary caries, and periodontal issues. This research sought to evaluate the vertical marginal precision of lithium disilicate crowns fabricated via the traditional heat-press methodology, the computer-aided design-milled wax pattern subsequently subjected to heat-pressing, and the fully digital computer-aided design/computer-aided manufacturing (CAD/CAM) milling of lithium disilicate materials.

Materials and methods

Thirty lithium disilicate crowns were fabricated on an identical master die of a prepared maxillary left second premolar and equally divided into three groups (n = 10): Group 1, conventional heat press using manually waxed patterns (control); Group 2, heat-press using CAD-milled wax patterns; and Group 3, fully digital CAD/CAM milling of lithium disilicate blocks (IPS e.max CAD LT A2, Ivoclar Vivadent, Schaan, Liechtenstein) followed by crystallization firing. All procedures were performed by a single operator. Vertical marginal gaps were measured at four indexed points using a stereomicroscope at 20x magnification (Leica M205 C; Leica Microsystems GmbH, Wetzlar, Germany). Data were then analyzed (α = 0.05).

Results

Significant intergroup differences were observed (p = 0.0001). Group 3 (fully digital) exhibited the smallest mean marginal gap, followed by Groups 2 and 1. Post-hoc analysis confirmed significant differences between all pairs on the mesial, distal, and palatal surfaces (p < 0.05). On the labial surface, Group 3 remained superior to Group 2 (p = 0.0021); however, no significant difference was noted between Groups 1 and 3 (p = 0.8739).

Conclusion

Fully digital CAD/CAM milling of lithium disilicate provided superior vertical marginal accuracy compared to heat-pressed techniques. Incorporating CAD-milled wax patterns significantly improved the fit compared to conventional manual waxing.

## Introduction

Restoration of damaged teeth with all-ceramic crowns has transformed prosthodontics, offering superior esthetics, biocompatibility, and durability compared to traditional metal-ceramic systems. As patient expectations for natural-looking, long-lasting restorations increase, the precision of crown fabrication has become paramount [1]. A critical determinant of clinical success is the marginal adaptation of the crown. A previous study proposed an average marginal range of 50-150 μm [2], whereas other studies advocate a range of 200-300 μm as being more advantageous [3,4]. A poor marginal fit can lead to microleakage, plaque accumulation, and secondary caries, ultimately compromising restoration longevity.

Advancements in dental materials and manufacturing technologies have expanded the treatment options. Computer-aided design/computer-aided manufacturing (CAD/CAM) systems enable a digital workflow from impression to milling, producing high-strength ceramic restorations with consistent accuracy [5]. Conversely, the heat-press technique, which utilizes lost-wax and high-temperature pressing of lithium disilicate or similar ceramics, remains a reliable conventional method valued for its adaptability and esthetic outcomes [6]. While both techniques aim to minimize marginal discrepancies, variations in processing, such as material shrinkage, milling precision, and operator influence, may affect the fit. Earlier research has yielded inconsistent outcomes regarding the marginal accuracy of crowns produced through CAD/CAM methodologies in comparison to those created via the heat pressing technique [7,8]. A previous study reported the lowest vertical marginal gap distance with the heat-press technique using CAD/CAM wax patterns [7], whereas another study reported no significant differences in marginal adaptation of all-ceramic crowns fabricated with CAD/CAM or the heat-press technique. Therefore, comparative data on vertical marginal accuracy between CAD/CAM-milled and heat-pressed all-ceramic crowns remain inconsistent, necessitating targeted in vitro evaluation. This study aimed to evaluate the vertical marginal gap of all-ceramic posterior crowns produced by CAD/CAM and heat press techniques, and to determine differences in marginal adaptation between conventional heat press, CAD/CAM-wax and heat press, and CAD/CAM lithium disilicate crowns.

## Materials and methods

Study design and setting

This controlled in vitro experimental study was conducted in the Department of Prosthodontics, Dasmesh Institute of Research and Dental Sciences, Faridkot, Punjab, India, for a period of six months. This study utilized a standardized typodont model to ensure reproducibility and eliminate biological variability. Since no human or animal subjects were involved, ethical approval was not required.

Sample size and grouping

The sample size was determined using the G*Power software (version 3.1.9.2; Heinrich Heine University, Düsseldorf, Germany). A total of 10 tooth crowns per group (total of 3 groups) were feasible for the analysis of marginal gap (mm) to achieve 80% power and 5% alpha error. An effect size of 0.6 was derived from a prior investigation concerning the marginal adaptation of pressed and CAD/CAM lithium disilicate dental crowns [9]. A total of 30 all-ceramic crowns were fabricated on a standardized master die and randomly allocated into three groups (n=10 per group): Group 1 (control), conventional heat press technique; Group 2, CAD-wax and heat press technique; and Group 3, CAD/CAM lithium disilicate (fully digital).

Specimen preparation

The maxillary left second premolar (tooth 25), in a typodont model (Frasco GmbH, Germany), was prepared for all-ceramic crown restoration. A removable metallic index die of the prepared tooth was fabricated and indexed at four reference points: mid-buccal, mid-palatal, mid-mesial, and mid-distal. This ensured consistent crown seating and standardized measurement locations. All fabrication procedures were performed by a single operator to minimize inter-operator variability.

Group 1: Conventional Heat Press Technique (Control)

Using a customized impression tray, 10 conventional impressions were made of the master die using the addition silicone impression material. Full-contoured wax patterns were fabricated on stone casts using modeling wax for pressable ceramics (Bego, Bremen, Germany) via the wax addition technique. Ten lithium disilicate crowns were pressed using ingots (IPS e.max Press LT, Ivoclar Vivadent) in an automatic press furnace (Programat EP5000, Ivoclar Vivadent), according to the manufacturer’s recommended protocol.

Group 2: CAD-Wax and Heat Press Technique

Ten anatomical CAD wax patterns were milled using high-density milling wax in a milling machine (Roland DWX-50 5-axis; Roland DG Corp., Japan). The milled wax patterns were then invested and heat-pressed into lithium disilicate crowns (IPS e.max Press LT, Ivoclar Vivadent) using an automatic press furnace under the same protocol as in Group 1.

Group 3: CAD/CAM Lithium Disilicate (Fully Digital)

Ten crowns were directly milled from lithium disilicate blocks (IPS e.max CAD LT A2, Ivoclar Vivadent, Schaan, Liechtenstein) using a five-axis milling unit (Cutter ME-300HP, Turbo Dent System), as to the manufacturer’s instructions. The milled restorations underwent crystallization firing in a ceramic furnace, according to the manufacturer’s guidelines.

Post-fabrication processing

All crowns were divested, and the reaction layer was removed. Sprues were sectioned, and minor occlusal adjustments were made. Internal adaptation was verified using a fit-checking silicone (Fit Checker, GC Corporation). No glazing or external staining was applied to avoid influencing the marginal fit. The crowns were preserved in distilled water at a temperature of 37 °C for a duration of 24 hours before assessment.

Measurement of the vertical marginal gap

**Figure 1:**
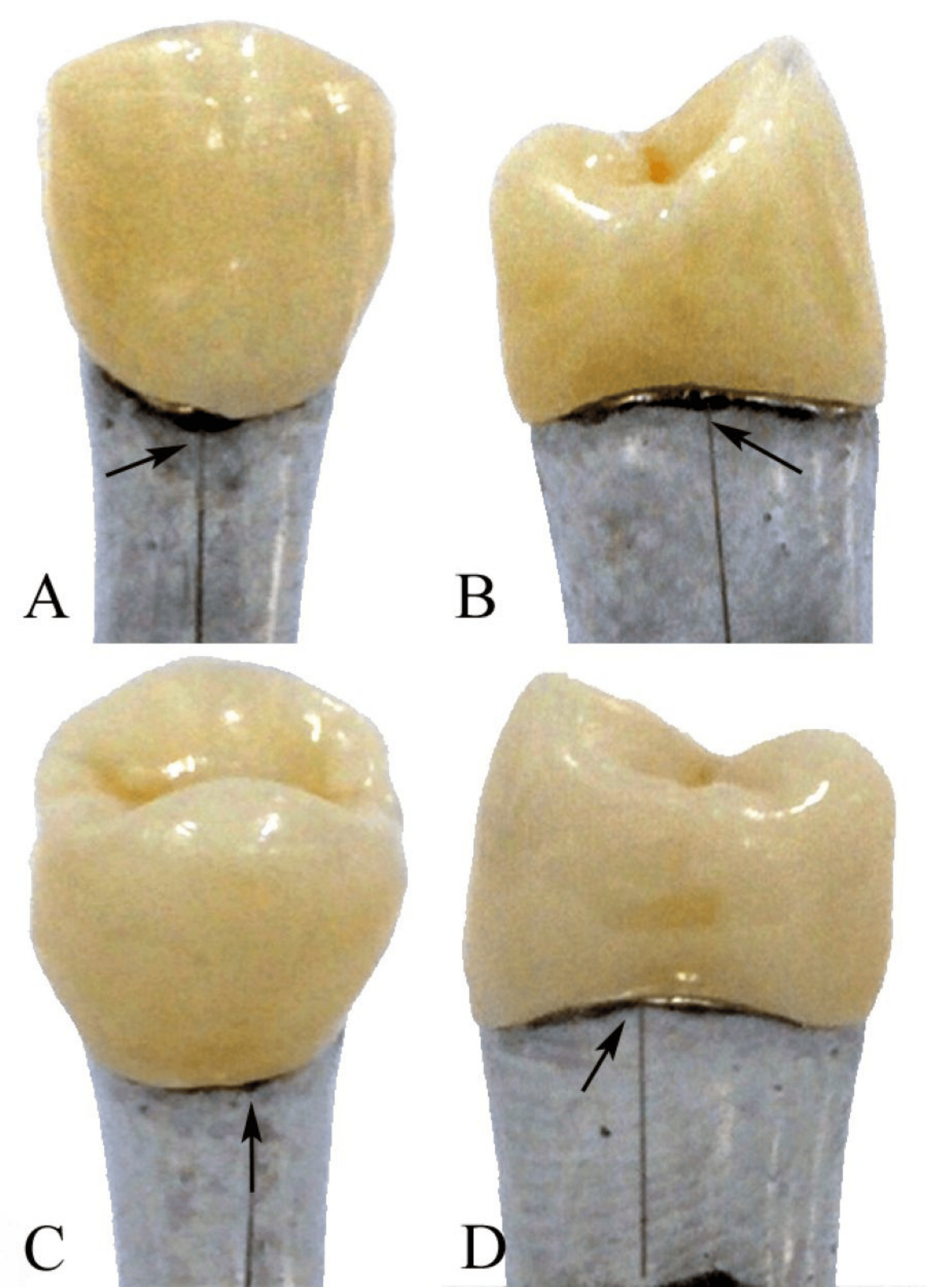
Representative stereomicroscopic images showing vertical marginal gap measurements at four standardized reference points on the maxillary left second premolar master die: (A) mid-buccal, (B) mid-mesial, (C) mid-palatal, and (D) mid-distal surfaces. Arrows indicate the vertical distance between the crown margin and the finish line of the prepared tooth. All measurements were performed under a stereomicroscope at 20× magnification using a metallic index to ensure reproducible positioning. Original image from the study samples.

The vertical marginal gap was assessed at four predetermined reference locations (mid-buccal, mid-palatal, mid-mesial, and mid-distal) utilizing a metallic index to ensure consistent positioning. The measurements were conducted under a stereomicroscope at a magnification of 20x (Leica M205 C; Leica Microsystems GmbH, Wetzlar, Germany). The average vertical marginal gap for each specimen was computed (Figure 1).

Statistical analysis

The data were subjected to analysis utilizing the Statistical Package for Social Sciences (SPSS) version 23.0 (IBM Corp., Armonk, New York, USA). The marginal gap measurements (mm) that were recorded were systematically organized employing Microsoft Excel (Microsoft Corporation, Redmond, WA, US). The normality of the data distribution was assessed through the Shapiro-Wilk test. Subsequently, intergroup comparisons for each aspect of the tooth were executed using one-way analysis of variance (ANOVA), followed by post-hoc pairwise comparisons via Tukey's test, with a significance threshold established at p < 0.05.

## Results

As shown in Table 1, the intergroup comparison of the marginal gap across all crown aspects of the maxillary left second premolar typodont tooth revealed highly statistically significant differences (p = 0.0001). Group 3 consistently demonstrated the smallest marginal gaps, followed by Group 2, whereas Group 1 showed the largest gaps. The pattern was uniform across the mesial, distal, labial, and palatal surfaces.

**Table 1:** Intergroup comparison of marginal gap (mm) on all aspects of the maxillary left second premolar crown among the study groups CAD/CAM: computer-aided design/computer-aided manufacturing *p = 0.0001 denotes high statistical significance using one-way analysis of variance (ANOVA). Data on the marginal gap are presented as mean ± standard deviation in millimeters (mm).

Crown aspects of maxillary left second premolar	Conventional heat press technique (Group 1, control)	CAD-wax and heat press technique (Group 2)	CAD/CAM lithium disilicate (Group 3)	F stats	p-value
Mesial	0.11 ± 0.005	0.07 ± 0.032	0.03 ± 0.003	97.5	0.0001*
Distal	0.11 ± 0.002	0.09 ± 0.002	0.05 ± 0.004	59.9	0.0001*
Labial	0.10 ± 0.003	0.06 ± 0.004	0.03 ± 0.003	224.6	0.0001*
Palatal	0.11 ± 0.003	0.08 ± 0.004	0.04 ± 0.004	71.2	0.0001*

As shown in Table 2, intragroup pairwise comparisons revealed a distinct pattern. For the mesial, distal, and palatal aspects of the maxillary left second premolar typodont tooth, all material pairings showed statistically significant differences (p < 0.05). However, on the labial aspect, the comparisons between Group 1 and Group 2 as well as Group 1 and Group 3 were not significant (p = 0.2869 and p = 0.8739, respectively), whereas the comparison between Group 2 and Group 3 remained significant (p = 0.0021). The inference is that while the material type significantly influences the marginal gap on most surfaces, the labial aspect shows a unique behavior, where the conventional heat-press method is not statistically distinguishable from other techniques.

**Table 2:** Intragroup pairwise comparison of marginal gap (mm) on all aspects of the maxillary left second premolar typodont tooth crown (mean differences analyzed using Tukey’s post-hoc test) *p < 0.05 denotes statistical significance using post-hoc Tukey’s test. CAD/CAM: computer-aided design/computer-aided manufacturing

Pairwise group	Mesial		Distal		Labial		Palatal	
	T stats	p-value	T stats	p-value	T stats	p-value	T stats	p-value
Conventional heat press technique vs. CAD-wax and heat press technique	3.91	0.0002*	22.37	0.0001*	25.29	0.2869	18.97	0.0001*
Conventional heat press technique vs. CAD/CAM lithium disilicate	43.38	0.0001*	42.42	0.0001*	52.17	0.8739	44.27	0.0001*
CAD-wax and heat press technique vs. CAD/CAM lithium disilicate	3.93	0.0002*	28.28	0.0001*	18.97	0.0021*	22.36	0.0001*

## Discussion

This in vitro study evaluated and compared the vertical marginal gap of lithium disilicate all-ceramic crowns fabricated using three different workflows: the conventional heat-press technique, CAD-milled wax followed by heat pressing, and fully digital CAD/CAM milling of lithium disilicate blocks. The results demonstrated statistically significant differences among the groups, with CAD/CAM-milled lithium disilicate crowns exhibiting the smallest marginal gaps, followed by CAD-wax/heat-pressed crowns, whereas conventionally pressed crowns showed the largest discrepancies. These findings highlight the influence of the fabrication technique and workflow precision on the marginal adaptation of all-ceramic restorations.

The superior performance of CAD/CAM lithium disilicate may be attributed to the digital workflow’s reduced number of procedural steps and minimized operator-dependent variability. CAD/CAM milling eliminates the wax pattern fabrication stage and multiple laboratory procedures in which distortion, thermal contraction, or human error can occur [10]. The milling of pre-crystallized lithium disilicate blocks provides a more dimensionally stable substrate, and the subsequent crystallization firing introduces consistent controlled expansion across the specimens. These explanations align with the observations reported by Reich et al., who found that CAD/CAM-milled lithium disilicate all-ceramic crowns showed good marginal accuracy with all CAD/CAM systems owing to fewer cumulative distortions during fabrication [11]. Similarly, Anadioti et al. reported a superior marginal fit for milled lithium disilicate crowns compared with pressable ceramics, supporting the trend identified in the present study [9]. Nevertheless, an earlier investigation indicated that the technique of press fabrication yielded a more favorable internal fit of onlays in comparison to the CAD/CAM method [11].

The CAD-wax and heat-press techniques demonstrated improved marginal adaptation compared to conventional heat-pressing, indicating the benefit of using digitally milled wax patterns over manually fabricated full-contour wax-ups. Conventionally created wax patterns are highly sensitive to operator skill, wax handling, thermal changes, and investment. Variations during waxing can lead to uneven shrinkage or distortion prior to investment, ultimately affecting the final crown’s marginal fit. Conversely, CAD-wax milling produces standardized patterns with high dimensional accuracy and eliminates the challenges associated with manual waxing [12]. These results concur with findings from previous studies, which observed that CAD/CAM-milled wax patterns significantly reduced marginal discrepancies when compared to conventional waxing in pressable ceramic systems [13,14].

The conventional heat press technique showed the largest marginal gaps, which can be explained by the inherent limitations of conventional multistep procedures. Impression-making, wax-up, investing, pressing, and divesting introduce potential sources of error. Distortion during impression removal, wax deformation, or inaccuracies in burnout and pressing can cumulatively contribute to larger discrepancies 12]. This outcome is consistent with a previous study that reported that conventionally pressed ceramic crowns tend to have larger marginal discrepancies than CAD/CAM-fabricated restorations because of greater technique sensitivity [15]. In contrast, Zeltner et al. reported no significant differences between the digital and conventional techniques [16].

Although Group 3 showed the smallest gaps across most surfaces, the labial aspect showed no statistically significant difference between Groups 1 and 3. This localized behavior may be due to the morphology of the premolar, the direction of die insertion, or minor seating variations during measurement. The labial surface is generally more accessible and less susceptible to distortion during investment or milling, potentially reducing intergroup differences.

Overall, all three groups demonstrated clinically acceptable marginal gaps based on established thresholds. McLean and von Fraunhofer [17] recommended an upper limit of 120 μm for clinical acceptability, whereas other authors suggested that discrepancies below 150 μm are unlikely to compromise restoration longevity [18]. The values obtained in this study fell within these ranges, indicating that all workflows can produce clinically usable restorations when carefully executed. However, the significantly superior performance of the fully digital CAD/CAM group emphasizes the advantages of modern digital manufacturing in achieving an optimal fit.

Clinical implications

The findings of this study have important implications for routine prosthodontic practice. First, the CAD/CAM milling of lithium disilicate crowns offers superior marginal accuracy and eliminates the technique-sensitive steps inherent to conventional methods. Clinicians adopting fully digital workflows can expect improved consistency, reduced laboratory errors, and potentially longer restoration lifespans owing to reduced cement dissolution and microleakage. Second, CAD/CAM-milled wax patterns provide a viable intermediate solution for centers transitioning toward full digital adoption. They enhance precision, even when final pressing is required for esthetic or material reasons. Finally, although conventional pressing remains a reliable technique, practitioners must be aware of its susceptibility to distortions and ensure strict procedural standardization.

Limitations

The study is limited by its in vitro nature and the use of a typodont model, which does not precisely replicate intraoral conditions such as thermal fluctuations, saliva contamination, masticatory forces, and periodontal ligament compliance. Only one tooth preparation design and one ceramic system were evaluated, which may limit generalizability. Additionally, the measurement of marginal gaps at four points may not entirely capture the circumferential variability, although this method is widely accepted. Future studies should include micro-computed tomographic analysis, thermomechanical aging, evaluation of different CAD/CAM systems, and clinical trials to validate the long-term performance.

## Conclusions

This in vitro study revealed that fully digital milling of posterior lithium disilicate crowns achieved the best vertical marginal adaptation, followed by a hybrid approach of computer-designed wax patterns with heat pressing. Conventional heat pressing consistently produced the largest marginal discrepancies owing to multiple technique-sensitive steps. Although all methods yielded clinically acceptable results, the digital and digitally assisted workflows demonstrated superior precision and reproducibility. Surface-specific variations further underline the impact of preparation geometry on the marginal accuracy. These findings support the preferential use of digital or hybrid digital techniques for fabricating lithium disilicate restorations in contemporary prosthodontic practice.
